# Case Report: Post-COVID-19 Vaccine Recurrence of Guillain–Barré Syndrome Following an Antecedent Parainfectious COVID-19–Related GBS

**DOI:** 10.3389/fimmu.2022.894872

**Published:** 2022-07-18

**Authors:** Margherita Bellucci, Francesco Germano, Stefano Grisanti, Chiara Castellano, Francesco Tazza, Emanuela Maria Mobilia, Davide Visigalli, Giovanni Novi, Federico Massa, Silvia Rossi, Paolo Durando, Corrado Cabona, Angelo Schenone, Diego Franciotta, Luana Benedetti

**Affiliations:** ^1^ Department of Neuroscience, Rehabilitation, Ophthalmology, Genetics, Maternal and Child Health, University of Genova, Genova, Italy; ^2^ IRCCS, Ospedale Policlinico San Martino, Genova, Italy

**Keywords:** COVID-19 vaccine, ganglioside antibodies, Guillain–Barrè syndrome, autoimmune diseases, case report

## Abstract

Guillain–Barré syndrome (GBS) is an autoimmune neurological disorder often preceded by viral illnesses or, more rarely, vaccinations. We report on a unique combination of postcoronavirus disease 2019 (COVID-19) vaccine GBS that occurred months after a parainfectious COVID-19–related GBS. Shortly after manifesting COVID-19 symptoms, a 57-year-old man developed diplopia, right-side facial weakness, and gait instability that, together with electrophysiology and cerebrospinal fluid examinations, led to a diagnosis of post-COVID-19 GBS. The involvement of cranial nerves and IgM seropositivity for ganglioside GD1b were noteworthy. COVID-19 pneumonia, flaccid tetraparesis, and autonomic dysfunction prompted his admission to ICU. He recovered after therapy with intravenous immunoglobulins (IVIg). Six months later, GBS recurred shortly after the first dose of the Pfizer/BioNTech vaccine. Again, the GBS diagnosis was confirmed by cerebrospinal fluid and electrophysiology studies. IgM seropositivity extended to multiple gangliosides, namely for GM3/4, GD1a/b, and GT1b IgM. An IVIg course prompted complete recovery. This case adds to other previously reported observations suggesting a possible causal link between SARS-CoV-2 and GBS. Molecular mimicry and anti-idiotype antibodies might be the underlying mechanisms. Future COVID-19 vaccinations/revaccinations in patients with previous para-/post-COVID-19 GBS deserve a reappraisal, especially if they are seropositive for ganglioside antibodies.

## Introduction

Guillain–Barré syndrome (GBS), a rare autoimmune-mediated polyradiculoneuropathy that has infectious episodes and vaccinations as triggers, can cause severe disability in up to 14% of patients, with a 1-year mortality rate estimated at between 3% and 20% (1). A lot of reports link coronavirus disease 2019 (COVID-19) to GBS, but the underlying pathophysiological correlations are not proved at present (2) or considered still pending at least (3). Currently, and in a future perspective, the mass vaccination campaign against severe acute respiratory syndrome coronavirus 2 (SARS-CoV-2), unprecedented in terms of scale and pace, is shifting the interest in such correlations, primarily between GBS and the COVID-19 vaccine.

GBS has been associated with vaccines against viruses in very rare cases, and the evidence of association with the COVID-19 vaccine is lacking but warrants further studies (4). Woo and colleagues, calculating the observed-to-expected ratio of postvaccine GBS, raised potential safety concerns for GBS following receipt of the Ad26.COV2.S COVID-19 vaccine (5). Furthermore, among the rare neurological complications of the COVID-19 vaccines, the mRNA vaccine ChAdOx1 nCov-19 was found to increase the risk of GBS, a finding confirmed in a second cohort (6). Finally, analyzing all cases of post-COVID-19 vaccine GBS reported to the WHO pharmacovigilance database, Pegat and colleagues noticed that GBS occurring in people vaccinated with adenovirus-vectored vaccines can present a specific and unusual phenotype characterized by facial paralysis, which might support a causal relationship between the vaccines and GBS (7). This phenotype, however, can follow COVID-19 with similar frequency (3). The main limitations of these studies include the passive reporting systems and presumptive case definition biases. In general, unusual adverse events, such as vaccine-induced immune thrombotic thrombocytopenia, myocarditis, and IgA vasculitis could also support potential links to COVID-19 vaccines (8).

We report on a patient with post-COVID-19 vaccine GBS who had previously had a COVID-19–related GBS with seropositivity for ganglioside IgM. This unique combination of events suggests that SARS-CoV-2 infection could have triggered, in a genetically predisposed subject, peripheral nerve-specific autoimmunity.

## Case Description

In April 2021, a 57-year-old man, with a history of Bell’s palsy 10 years earlier, was admitted to our Neurology Department with a 5-day–lasting moderate fever and arthromyalgia, followed by diplopia, right-side facial weakness, and gait instability. Nasopharyngeal swab PCR and antigenic tests for SARS-CoV-2 were positive. Chest CT imaging findings were typical of COVID-19 pneumonia. Antiviral therapy with remdesivir (IV, 200 mg loading dose on day 1, followed by a 100-mg maintenance dose administered daily on days 2 through 10) was started. Neurological examination revealed right-side third and seventh cranial nerve palsy, distal weakness in four limbs (Medical Research Council (MRC) scale for muscle strength scores: wrist extensors, 4/5 bilaterally; interossei, 3/5 bilaterally; extensor hallucis longus, 3/5 on the right and 4/5 on the left; extensor digitorum longus, 4/5 bilaterally), bilateral stocking hypoesthesia, gait ataxia, and global areflexia. Cerebrospinal fluid (CSF) analysis disclosed albuminocytological dissociation, a diagnostic hallmark of GBS (**Table 1**). Electrophysiology studies confirmed the diagnosis, showing features consistent with acute demyelinating sensory-motor polyneuropathy (**Table 2**). Serum IgM to the ganglioside GD1b were positive on both immunoblot (Alifax, Padua, Italy) and ELISA (Bühlmann, Schönenbuch, Switzerland). Immunoblots for serum IgG and IgM to GM1/2/3/4, GD1a/b (except for GD1b IgM), GD2/3, GT1a/b, GQ1b, and sulfatides were all negative, as was ELISA for GD1b IgG, GM1 and GQ1b IgG, and IgM.

**Table 1:** Patient’s clinic and paraclinic features of the first (post-COVID-19 GBS) and of the second event (post-COVID-19 vaccine GBS recurrence).

		GBS type	CSF analysis		Ganglioside antibodies (IgM)	Additional features		Therapy	Disability scales	
			Ly (/µL)*	Prot (g/L)^		Cranial nerve	Autonomic dysfunction		MRC-MS (T0; T1)	GBS-DS (T0; T1)
**1^st^ event**		AIDP	2	0.58	GD1b	yes	yes	IVIg	24; 60	4; 0
**2^nd^ event**		AIDP	<2	0.81	GM3/4, GD1a/b, GT1b	no	no	IVIg	54; 60	3; 0

GBS, Guillain-Barré syndrome; CSF, cerebrospinal fluid; Ly, lymphomonocytes; *reference range, < 5; Prot, total proteins; ^reference range, < 0.52; MRC-MS, Medical Research Council Muscle Score; GBS-DS, Guillain Barré Syndrome Disability Score; AIDP, Acute Inflammatory Demyelinating Polyneuropathy; IVIg, intravenous immunoglobulins; T0, neurological evaluation at onset; T1, neurological evaluation at 3-month follow-up.

**Table 2:** Electrophysiology performed during the first (post-COVID-19 GBS) and second events (post-COVID-19 vaccine GBS recurrence).

Nerve conduction studies						
	Right motor nerves			Right sensory nerves		
	DML (ms)	MCV (m/s)	cMAP (mV)		SCV (m/s)	SAP (µV)
**1st event**						
*Ulnar*				*Ulnar*		
Wrist	**4.8** (<3.0)		**2.3** (>2.8)	Wrist	**NE** (>44.0)	**NE** (>18.0)
Below elbow	**10.3** (<3.0)	**36.7** (>49.0)	**1.6** (>2.8)			
Above elbow	**15.4** (<3.0)	**23.5** (>50.0)	**1.0** (>2.8)			
*Peroneal*				*Sural*		
Ankle	**12.7** (<5.5)		**0.8** (>2.5)	Calf	**30.6** (>40.0)	**2.1** (>5.0)
Fibular head	**21.9** (<5.5)	**34.6** (>40.0)	**0.5** (>2.5)			
Knee	**26.9** (<5.5)	**24.0** (>40.0)	**0.3** (>2.5)			
**2nd event**						
*Ulnar*				*Ulnar*		
Wrist			**8.3** (>2.8)	Wrist	**NE** (>44.0)	**NE** (>18.0)
Below elbow		**29.0** (>49.0)	**4.9** (>2.8)			
Above elbow	**13.2** (<3.0)	**44.0** (>50.0)	**4.7** (>2.8)			
*Peroneal*				*Sural*		
Ankle	**5.0** (<5.5)		**2.3** (>2.5)	Calf	**NE** (>40.0)	**NE** (>5.0)
Fibular head	**13.0** (<5.5)	**35.0** (>40.0)	**0.5** (>2.5)			
Knee	**16.5** (<5.5)	**26.0** (>40.0)	**0.4** (>2.5)			

cMAP, compound muscle action potential; DML, distal motor latency; MCV, motor conduction velocity; SAP, sensory action amplitude; SCV, sensory conduction velocity; NE, nonexcitable; ms, milliseconds; m/s, meter/second; mV, millivolt; µV, microvolt. Normal reference values are reported in round brackets; abnormal values are reported in bold character.

Recent infections that more commonly precede GBS were reasonably excluded by negative results for *Cytomegalovirus* IgM, *Mycoplasma pneumoniae* IgM, *Campylobacter jejuni* IgM/IgA, *Epstein–Barr virus* capsid antigen (VCA) IgM, and *Haemophilus influenzae* IgM. Rapid worsening of breathing pattern and neurological conditions, characterized by asymmetrical flaccid tetraparesis (MRC scores: 3/5 in the left upper limb and 1/5 in the right lower limb) and autonomic dysfunction with profuse sweating, led to admission to ICU, where he was treated with high-flow oxygen therapy only. A course of IVIg (0.4 g/kg for 5 days) prompted gradual progressive functional recovery, which became complete after neurorehabilitation. As such, a follow-up electrophysiology study was not performed.

In October 2021, the patient received the first dose of the BNT162b2 (Pfizer/BioNTech) mRNA vaccine and had a moderate fever for 2 days. Five days later, he developed disequilibrium, difficulty climbing stairs, and foot hypoesthesia. He was soon readmitted to our Neurology Department. Neurological examination showed areflexia, lower limb weakness (MRC scale scores: extensor hallucis longus, 3/5 on the right; 4/5 on the left; extensor digitorum longus, 4/5 on the right), ataxic gait, and need of support for walking. Albuminocytological dissociation was found in CSF analysis, and electrophysiology studies confirmed a demyelinating sensory-motor polyneuropathy (**Tables 1**, **2**). The extensive screening for recent infections by the most commonly GBS-associated infectious agents was repeated, resulting negative as well. The immunoblot for ganglioside antibodies was positive for GM3/4, GD1a/b, and GT1b IgM (the latter confirmed on ELISA). Serum agarose electrophoresis excluded a monoclonal gammopathy. After only 5 days from vaccination, the patient showed strong SARS-CoV-2 serological responses both to the vaccine (receptor-binding domain (RBD) antibodies, 15,193.0 U/ml; reference values, <0.8; Roche) and in terms of the natural immunity (nucleocapsid antibodies, 95.6 ICO; reference values <1.0; Roche). A new course of IVIg (0.4 g/kg for 5 days) prompted complete recovery, as assessed at a 4-month follow-up when only weak seropositivity for GT1b IgM persisted. No adverse effects occurred over the two IVIg courses.

## Discussion and Conclusions

Our patient’s first GBS had the features of a parainfectious event due to the shorter than the 2-week interval between infection and GBS onset, in line with what was reported in Zika virus (9) and in other SARS-CoV-2–associated GBS (10; personal communication). Indeed, 2–3 weeks is the typical time frame between infection and disease onset required by the humoral immune response to develop in postinfectious GBS (9). As in Zika virus–associated GBS (9), the electrophysiological features of both COVID-19 infection– and COVID-19 vaccination–associated GBS in our patients were suggestive of acute inflammatory demyelinating polyneuropathy.

Seropositivity for ganglioside antibodies has been only occasionally detected in COVID-19–associated GBS (5, 6, 10). In our patient, the post-COVID-19 vaccine GBS was characterized by a previously unreported combination of IgM to GD1b and other disialosyl gangliosides. These antibodies are unusually present in typical GBS, while they may be occasionally present in ataxic forms, such as Chronic Ataxic Neuropathy Ophthalmoplegia IgM paraprotein Cold Agglutinins Disialosyl antibodies (CANOMAD) and Chronic Ataxic Neuropathy with Disialosyl Antibodies (CANDA) (11) and may be the result of autoimmunity elicited by viral epitopes cross-reacting with gangliosides of the peripheral myelin sheaths (molecular mimicry). Furthermore, hyperstimulation of the immune system is a cofactor that could contribute to SARS-CoV-2–induced autoimmunity (12). Our patient showed strong SARS-CoV-2 serological responses both to the vaccine (RBD) and in terms of natural immunity after only 5 days from vaccination.

Outside the RBD, the N-terminal-domains (NTDs) of SARS-CoV-2 spike protein have been identified as supersites recognized by human NTD antibodies, some of which are able to neutralize SARS-CoV-2 ultrapotently (13). Fantini and colleagues suggested that the NTDs presumably recognize ganglioside binding sites that SARS-CoV-2 might use to bind to lipid rafts of plasma membranes alongside ACE-2 to enter the cells (14). Therefore, in our patient, anti-idiotype antibodies of the IgM class, possibly resulting from a T-cell–independent immune response, might have contributed to autoimmune-mediated phenomena initiated with the SARS-CoV-2 infection and reignited by the vaccine. These kinds of autoantibodies have been recently considered potential triggers of autoimmunity in COVID-19 and COVID-19 vaccines (15). Indeed, in these settings, it has been hypothesized that immune-cell attacks on ACE2-expressing cells could be responsible for the occurrence of myocarditis (15). In addition to ACE2, which is expressed on neuronal tissue too, the second receptor for SARS-CoV-2, neuropilin-1, might also specifically trigger autoimmunity against the peripheral nerves (16). Within this context, the possibility of IgM responses without class-switching to IgG is suggested by ACE-2 IgM autoantibodies in COVID-19 (17).

Other clues favoring the peripheral nerve–specific SARS-CoV-2–related autoimmune response in our patient include the COVID-19 vaccine–associated recurrence of GBS after an interval of many months without any symptoms; the fact that GBS, in general, relapses very rarely (2%–5%) (1); and the substantial negativization of the IgM response to multiple gangliosides at follow-up, after complete recovery. In a large population cohort, GBS recurrence after influenza revaccination occurred in no subjects with a history of GBS following previous influenza vaccination (18).

Our case should be contextualized as an extremely rare event but, in the light of future revaccination campaigns too, warns about GBS recurrence after COVID-19 vaccination and suggests that seropositivity for ganglioside antibodies could be a marker of this risk in patients with antecedent SARS-CoV-2–associated GBS.

In general, considering the huge number of individuals who will be either infected/reinfected with SARS-CoV-2 and its variants, or vaccinated/revaccinated for COVID-19 worldwide, reports of post-SARS-CoV-2 infection/COVID-19 vaccine GBS will increase in the near future. Even so, the causal relationship between these events and the development of GBS should be critically evaluated without the support of strong epidemiology and pathophysiology data (19).

## Patient Perspective

Our patient and his family were very concerned when, at the first event, the neurological symptoms manifested and even more so after the diagnosis of COVID-19 and his admission to ICU. The patient was relieved when informed that his neurological disease was treatable and that COVID-19 was not severe. He perceived a prompt improvement in his condition after IVIg therapy. He was extremely frustrated when the neurological disease occurred after COVID-19 vaccination but was confident that early therapy could be effective. IVIg treatment was rapidly efficacious. Eventually, he was thankful to the neurologists who had cured him and was glad to agree to share his case, understanding that he had had a very rare, if not exceptional, condition.

## Data Availability Statement

The original contributions presented in the study are included in the article/supplementary material. Further inquiries can be directed to the corresponding author.

## Ethics Statement

Written informed consent was obtained from the individual for the publication of any potentially identifiable images or data included in this article.

## Author Contributions

MB, FG, AS, DF and LB contributed to conception and design of the study. SG, ChC, FT, EM, DV, GN, FM, SR, PD and CoC contributed to data analysis. MB wrote the first draft of the manuscript. DF and LB revised the manuscript and produced its final version. All authors contributed to manuscript revision, read, and approved the submitted version. DF and LB contributed to the manuscript equally.

## Funding

This work received support from the Italian Ministry of Health under the grants “Fondi per la Ricerca Corrente 2021” and Rete delle Neuroscienze e della Neuroriabilitazione.

## Conflict of Interest

The authors declare that the research was conducted in the absence of any commercial or financial relationships that could be construed as a potential conflict of interest.

## Publisher’s Note

All claims expressed in this article are solely those of the authors and do not necessarily represent those of their affiliated organizations, or those of the publisher, the editors and the reviewers. Any product that may be evaluated in this article, or claim that may be made by its manufacturer, is not guaranteed or endorsed by the publisher.
